# Preparation and Performance of Multifunctional Self-Aggregating Modified Quartz Sand Proppant for Coalbed Methane Channel Fracturing

**DOI:** 10.3390/ma19173690

**Published:** 2026-08-30

**Authors:** Bin Wang, Mengqi Chen, Hongliang Lin, Zhifei Liang, Yong Ma, Yanfeng Yang, Ran Chen, Xiaoqin Pu

**Affiliations:** 1 Exploration and Development Construction Company, PetroChina Coalbed Methane Company Limited, Xi’an 710003, China; 2 College of Chemistry and Chemical Engineering, Chongqing University of Science and Technology, Chongqing 401331, China

**Keywords:** self-aggregating, quartz sand, proppant, coalbed methane

## Abstract

A multifunctional self-aggregation modified quartz sand support agent (MRQS) was prepared by a simple dry coating method for coal seam gas fracturing channels. The organic coating is composed of KH550, OTMS, H-PDMS and microcrystalline wax, which transforms the surface of the raw sand from super hydrophilic to hydrophobic (contact angle is 91.8°), thus providing a driving force for the spontaneous aggregation of particles in aqueous fluids. MRQS shows excellent anti-reflow stability, which does not dissipate after ultrasonic oscillation for more than 10 min, while raw sand can only withstand 3 min. Atomic force microscopy (AFM) analysis shows that the interface adhesion of MRQS under low load is tripled (106.7 nN vs. 32.0 nN), which explains its stronger aggregation intensity. In the temporary sealing test, MRQS forms a stable and low-permeability sealing layer at the narrow crack, raising the sealing pressure to 0.23 MPa, while the original sand cannot achieve effective sealing. The crack diversion ability test shows that MRQS still maintains a diversion capacity of 1.62 μm^2^·cm under a closed stress of 45 MPa, and the attenuation curve is gentle. Although the initial value is slightly lower than that of raw sand (1.88 μm^2^·cm), the overall performance is comparable. The static loss test shows that MRQS extends the filtration time of 200 mL filtrate to 439 s, which is 2.04 times that of the original sand. This simple dry coating strategy prepares a multifunctional support agent, which combines self-aggregation, temporary sealing, filter loss control and high stress stability, providing a practical solution to improve the fracturing performance of coal seam gas (CBM).

## 1. Introduction

Coal seam gas or coal bed methane (CBM) is an important unconventional clean energy source to optimize the global energy structure and ensure energy security [1,2]. China has abundant coal seam gas resources, but due to the inherent characteristics of reservoirs, such as ultra-low permeability, low stratum pressure and mainly adsorption gas, its efficient development is seriously constrained [3]. More than 70% of methane in the medium and shallow coal seams exists in an adsorbed state, resulting in a large number of untapped resources [4]. These characteristics have caused the “three low” development problems; that is, the degree of exploration is low, the output of a single well is small, and the resource recovery rate is low, thus limiting the exploitation of large-scale and economical coal seam gas [5,6,7].

Hydraulic fracturing is the main production-increasing technology for building artificial crack networks and connecting coal seam mezzanines and wellbores [8,9,10]. However, the weak and fragile characteristics of the coal seam reservoir itself will cause three major engineering problems of coupling, thus reducing the long-term effect of fracturing. First of all, the traditional quartz sand support agent is prone to serious embedding under locking stress, resulting in narrowing of the crack width and a decrease in the conduction performance. Guo et al. [11] confirmed through on-site tests that the original quartz sand conduction performance decayed rapidly, while the modified support agent showed more stable support performance. Secondly, fracturing backflow and airflow easily cause the backflow of the support agent, resulting in the failure of crack support. The complex coal seam sandwich further exacerbates the problem of particle migration. Ai et al. [12] pointed out that the residual coal powder will destroy the crack continuity, aggravate the leakage of the support agent, and weaken the effect of continuous production increase. Third, a large amount of movable coal powder will be produced when hydraulic fracturing intersects with natural cracks. Zhang et al. [13] showed that fissure bending and branching contribute to the formation of small cracks, while Jiang et al. [14] confirmed that low local stress difference will form complex fissure networks, which can be used as small migration channels. These problems are intertwined and fundamentally limit the long-term production capacity of coal seam gas wells.

Surface modification of low-cost quartz sand has become a mainstream strategy to improve proppant comprehensive performance [15,16]. Hydrophobic wettability modification effectively optimizes reservoir fluid flow. Wang et al. [17] prepared high-performance hydrophobic quartz sand with excellent water-plugging capacity, and Liu et al. [18] further developed composite nano-coatings to achieve more stable hydrophobicity and reservoir adaptability. Polymer coating technology solves proppant flowback defects. Differing from conventional one-time bonding resin proppants, Fu et al. [19] developed self-reaggregating polymer-modified proppants with outstanding anti-flowback durability under high stress. Krishnan et al. [20] fabricated graphene composite coated proppants with superior high-temperature and high-pressure resistance for deep reservoirs. Targeting coal fine damage, Li et al. [21] synthesized cementitious proppants with 96% high-efficiency coal fine blocking capacity, effectively protecting fracture seepage channels.

Despite these advances, existing modified proppants only achieve single-functional improvement. Few materials can simultaneously address the coupled challenges of proppant flowback, coal fine damage, severe fluid loss and fracture temporary plugging, restricting the further improvement of CBM fracturing efficiency. Notably, most hydrophobic modified proppants tend to sacrifice suspension and transport performance in water-based fracturing fluids, and their long-term interfacial stability and compatibility with complex fracturing fluid additives remain uncertain, which restricts their reliable application in practical CBM reservoir stimulation.

Channel fracturing, an innovative stimulation technology, constructs discrete proppant pillars and open flow channels to greatly enhance fracture conductivity, which is highly applicable for CBM development. Hou et al. [22] validated the superiority of heterogeneous proppant placement in improving reservoir flow capacity. Zheng et al. [23] clarified the core controlling factors of proppant pillars on fracture conductivity and provided field optimization guidance. Hulsurkar et al. [24] confirmed that modified proppants maintain more stable multiphase seepage characteristics, guaranteeing long-term channel flow stability. Nevertheless, channel fracturing application in CBM is still restricted by material bottlenecks. Luo et al. [25] optimized fracturing fluid performance to improve proppant transport capacity. Zhang et al. [26] proposed a micro-sealing agent strategy to reduce embedding damage under high stress. Elkhatib et al. [27] found that changes in interface wetting will lead to instability of the flow channel, while reasonable surface modification can maintain long-term conductivity. In general, the lack of multifunctional sealants with spontaneous self-aggregation ability is a key constraint to limit the development of CBM channel fracturing.

In response to the above research gaps, this work has developed a new multi-functional self-aggregation modified quartz sand support agent (MRQS), which is specially designed for coal seam gas channel fracturing through simple dry coating technology. The composite organic coating gives quartz sand good hydrophobicity without destroying the mineral skeleton. The prepared MRQS has the characteristics of spontaneous self-aggregation, temporary blockage, high-efficiency leakage prevention and high-stress conductivity stability, which comprehensively solves the coupling defects of traditional support agents. This study provides a feasible laboratory-scale modification strategy for high-performance multifunctional proppants, which shows promising application potential for stabilizing CBM fracture channels and improving gas recovery under laboratory test conditions.

## 2. Materials and Methods

### 2.1. Materials and Reagents

Raw quartz sand (RQS, 40–70 mesh) for industrial fracturing was used as the substrate. n-octyltrimethoxysilane (OTMS), hydrogen-terminated polydimethylsiloxane (H-PDMS), and microcrystalline wax (MCW) were purchased from Shanghai Macklin Biochemical Co., Ltd. (Shanghai, China). The silane coupling agents were of analytical grade, and H-PDMS was of industrial grade. Deionized water prepared in the laboratory was used for preparing all aqueous media. A custom-formulated slickwater fracturing fluid (0.4 wt.%) mimicking in situ coalbed reservoir conditions was used as the displacing medium.

### 2.2. Preparation of Modified Quartz Sand

**Figure 1:**
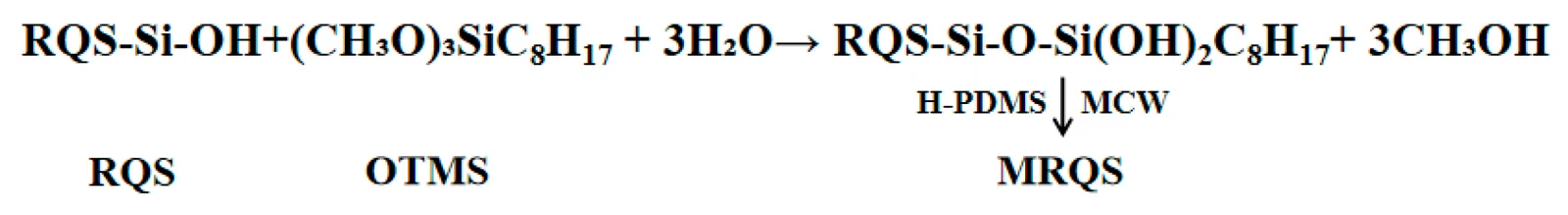
Schematic diagram of MRQS preparation.

A precision balance was used to weigh 100 g of RQS, which was then dried in a blast oven at 105 °C for 2 h. The dried sample was cooled to room temperature in a desiccator for later use. Subsequently, 0.1 g of OTMS were accurately weighed, mixed in a beaker under stirring at room temperature, and then transferred together with the pre-dried RQS into a high-speed mixer. The mixture was blended at high speed for 3–5 min. The frictional heat generated during high-shear stirring induced hydrolysis of the silanes (with water mainly derived from adsorbed moisture on the sand surface and ambient humidity) and subsequent condensation grafting onto the hydroxyl groups on the quartz sand surface. After discharging, the mixture was kept in a ventilated fume hood at an ambient temperature for 30 min to complete preliminary crosslinking and curing. The schematic diagram of MRQS preparation is shown in Figure 1.

Separately, 0.1 g of MCW was fully melted in a water bath at 90 °C, followed by addition of 0.01 g of H-PDMS under uniform stirring. The mixed auxiliary agent was then added dropwise into the silane-modified sand and blended at low speed for 2 min. After natural cooling, the multifunctional modified quartz sand proppant (denoted as MRQS) was obtained.

In this multi-step modification procedure, OTMS undergoes hydrolysis and covalently bonds to the quartz sand surface, serving as the primary hydrophobic modifier. H-PDMS further seals surface micro-pores of the silane-grafted sand to enhance surface hydrophobicity and enable self-aggregation via hydrophobic interactions in aqueous media. MCW adheres to the sand mainly through physical interactions; it fills residual interfacial pores originating from OTMS grafting, further improves hydrophobic performance, and reduces the overall modification cost.

### 2.3. Experimental Apparatus

Microscopic characterization instruments included a NICOLET iS10 Fourier-transform infrared spectrometer (Thermo Fisher, Waltham, MA, USA), a JSM-7800F field-emission scanning electron microscope (JEOL, Tokyo, Japan), and a Dimension Icon XR atomic force microscope (Bruker, Billerica, Germany). A SINDIN SDC-200S contact angle goniometer (SINDIN Intelligent Equipment Co., Ltd., Dongguan, China) was adopted to measure surface wettability.

For macroscopic performance evaluation, an NF-2 proppant fracture conductivity test system (Haian Petroleum Scientific Research Instrument Co., Ltd., Nantong, China) was used for fracture conductivity measurements in accordance with industry standard SY/T 6302-2019. Static fluid loss tests were performed on a GGS42-2A high-temperature and high-pressure filter press (Qingdao Ruisheng Tongda Technology Co., Ltd., Qingdao, China). A self-developed visual fracture simulation apparatus was employed for temporary plugging breakthrough pressure tests (Section 2.4.2), while a GDD-3 high-temperature high-pressure dynamic circulation plugging simulation device (Haian Petroleum Scientific Research Instrument Co., Ltd.) was used for relevant dynamic plugging characterization.

### 2.4. Macroscopic Performance Evaluation Methods

#### 2.4.1. Sand-Aggregation Strength Test

To quantitatively characterize the interfacial adhesion strength between proppant particles and to simulate the shear disturbance during fracturing flowback, an ultrasonic oscillation method was employed to compare the self-aggregation and anti-dispersion ability of RQS and MRQS.

(1) Sand-column preparation: A vertical transparent plastic tube with an inner diameter of 3 cm was used as the mold. For each test, 30 g of dry proppant (RQS or MRQS) was loaded into the tube. Slowly inject the deionized water into the tube wall until the particles are completely immersed, and then leave the chromatography column at room temperature for 10 min to allow self-aggregation. Then gently pour out the supernatant to avoid disturbing the structure of the column.
(2) Ultrasonic collapse test: Immerse the formed sand column into the ultrasonic cleaning tank together with its support net (40 kHz, constant power). Immediately start the ultrasonic oscillation and time it, and observe the peeling and crushing process throughout the whole process. Complete collapse is defined as the complete shedding of the sand column and no continuous particle residue. Repeat the test for each sample three times, and take the average collapse time as a quantitative indicator. If the sand column remains intact after 10 min of ultrasound treatment, the test will be stopped, and the recorded collapse time will be >10 min. The longer the collapse time, the stronger the interface bonding force, and the better the resistance to fluid flushing and filler backflow.

#### 2.4.2. Temporary Plugging Breakthrough Pressure Test

Using the self-developed visual fracture simulation device, the dynamic bearing capacity and water blocking performance of RQS and MRQS are compared. The simulated fracture has a wedge-shaped geometry with a wide inlet (2 mm) and a narrow outlet (1 mm), mimicking the actual reservoir fracture taper from the near-wellbore region to the distal tip. A total of 40 g of proppant was uniformly packed into the fracture cavity. The displacing fluid was the fracturing fluid containing 30 wt.% proppant. The test was conducted under constant-flow displacement. The system pressure and cumulative filtrate volume at the outlet were recorded in real time at various time points, allowing comparison of the two proppants in terms of plugging-layer formation efficiency, ultimate pressure-bearing capacity, and fluid-loss control.

#### 2.4.3. Static Fluid Loss Test

Static fluid-loss performance of fracturing fluid systems containing different proppants was evaluated following the industry standard SY/T 6376. The base fluid was a 0.4 wt.% guar gum aqueous solution prepared with deionized water. Three groups were compared: (1) blank group-pure guar base fluid without any proppant; (2) control group-base fluid mixed with 30 vol.% unmodified quartz sand (RQS); and (3) experimental group-base fluid mixed with 30 vol.% modified proppant (MRQS).

All fluid samples were dynamically aged at 70 °C for 4 h to simulate downhole reservoir conditions. Fluid-loss measurements were performed using a high-temperature, high-pressure static filter press equipped with HTP-1 filter paper (diameter = 63.5 mm). The test pressure differential was fixed at 3.5 MPa, and the cumulative filtrate volume was recorded every 10 s. The test was terminated when the cumulative filtrate volume reached 200 mL, and the corresponding filtration time for each group was recorded.

According to the classical constant-pressure filtration theory, the cumulative filtrate volume V follows a linear relationship with the square root of time: *V* = *k* · *t* ^0.5^, where *k* is the fluid-loss coefficient. Based on the measured data, *V* − *t* ^0.5^ curves were plotted for each group to quantitatively compare their fluid-loss control ability.

#### 2.4.4. Fracture Conductivity Measurement

Long-term fracture conductivity of the proppants was tested using a standard API conductivity cell. A fixed mass of 94 g of dry proppant was uniformly loaded into the cell. The displacing fluid was deionized water with a dynamic viscosity of 0.98 mPa·s at 25 °C. The designed confining-pressure gradient ranged from 0 to 45 MPa with a step of 5 MPa. Owing to equipment loading precision and compaction deformation of the proppant pack, slight deviations occurred in the actual confining pressure; therefore, the real-time measured pressure values were used in subsequent quantitative analysis.

The proppant-pack conductivity was calculated based on Darcy’s law using the following equation:

*W*_f_ = 5.555 × *μ* × *q*/Δ*P*

where *W*_f_ represents fracture conductivity (μm^2^·cm); *μ* denotes fluid viscosity (0.98 mPa·s); *q* is the flow rate of displacing fluid (mL/min); Δ*P* stands for the pressure difference between the inlet and outlet (kPa).

The confining pressure was increased stepwise. After the pressure and flow rate reached a steady state at each step, the pressure drop and flow velocity were recorded. The conductivity values of RQS and MRQS under various confining stresses were then calculated to compare their resistance to compaction and their ability to maintain effective flow channels.

#### 2.4.5. Atomic Force Microscopy (AFM) Test

Prior to AFM characterization, raw quartz sand was ball-milled to obtain fine particles suitable for microscopic scanning, and the fine particles were then adopted to prepare MRQS following the aforementioned dry coating procedure. The surface morphology and interparticle adhesion force of RQS and MRQS were measured using an AFM instrument. Topographic scanning was performed to acquire particle size and surface asperity height. Adhesion force measurements were conducted under set normal loads of 100 nN, 300 nN and 500 nN. Multiple random positions on each sample were tested, and the averaged values were adopted for quantitative comparison.

## 3. Results

### 3.1. Characteristic Analysis

**Figure 2:**
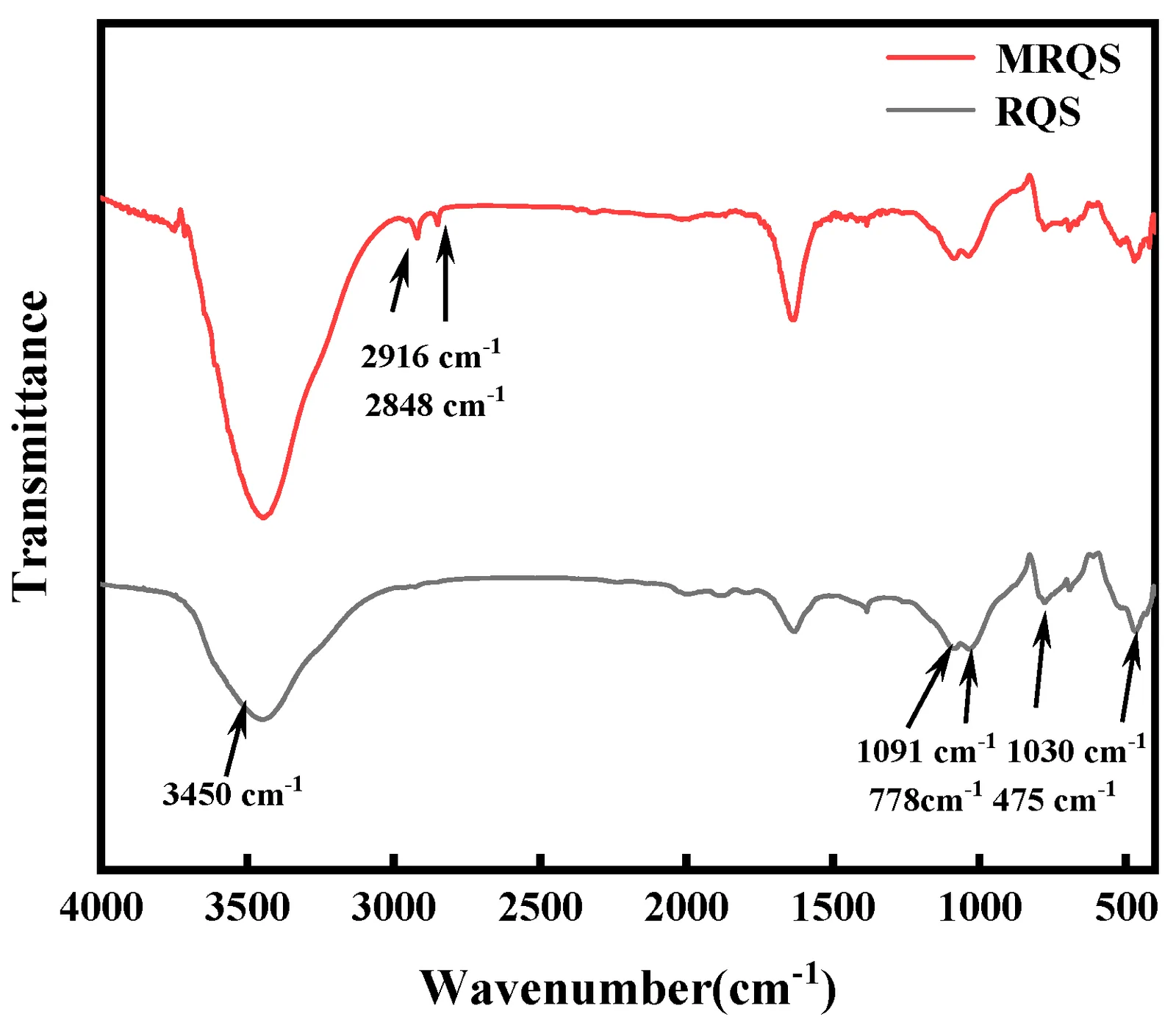
FTIR spectra of RQS and MRQS.

Figure 2 displays the FTIR spectra of RQS and MRQS. The broad band at 3450 cm^−1^, assigned to O-H stretching of silanol groups and adsorbed water, was drastically attenuated for MRQS, confirming effective shielding of hydrophilic Si-OH by the organic coating. Two characteristic peaks at 2916 cm^−1^ and 2848 cm^−1^ (C-H stretching of methylene and methyl groups) emerged exclusively for MRQS, directly verifying successful grafting of alkyl-rich modifiers. The fingerprint peaks of Si-O-Si (1091, 1030, 778 and 475 cm^−1^) remained basically unchanged, indicating that the quartz mineral skeleton was not damaged during the dry coating process. In general, the obvious presence of alkyl bands, the weakening of hydroxyl absorption capacity and the integrity of silicon dioxide peaks indicate the uniform distribution of organic coatings on MRQS.

**Figure 3:**
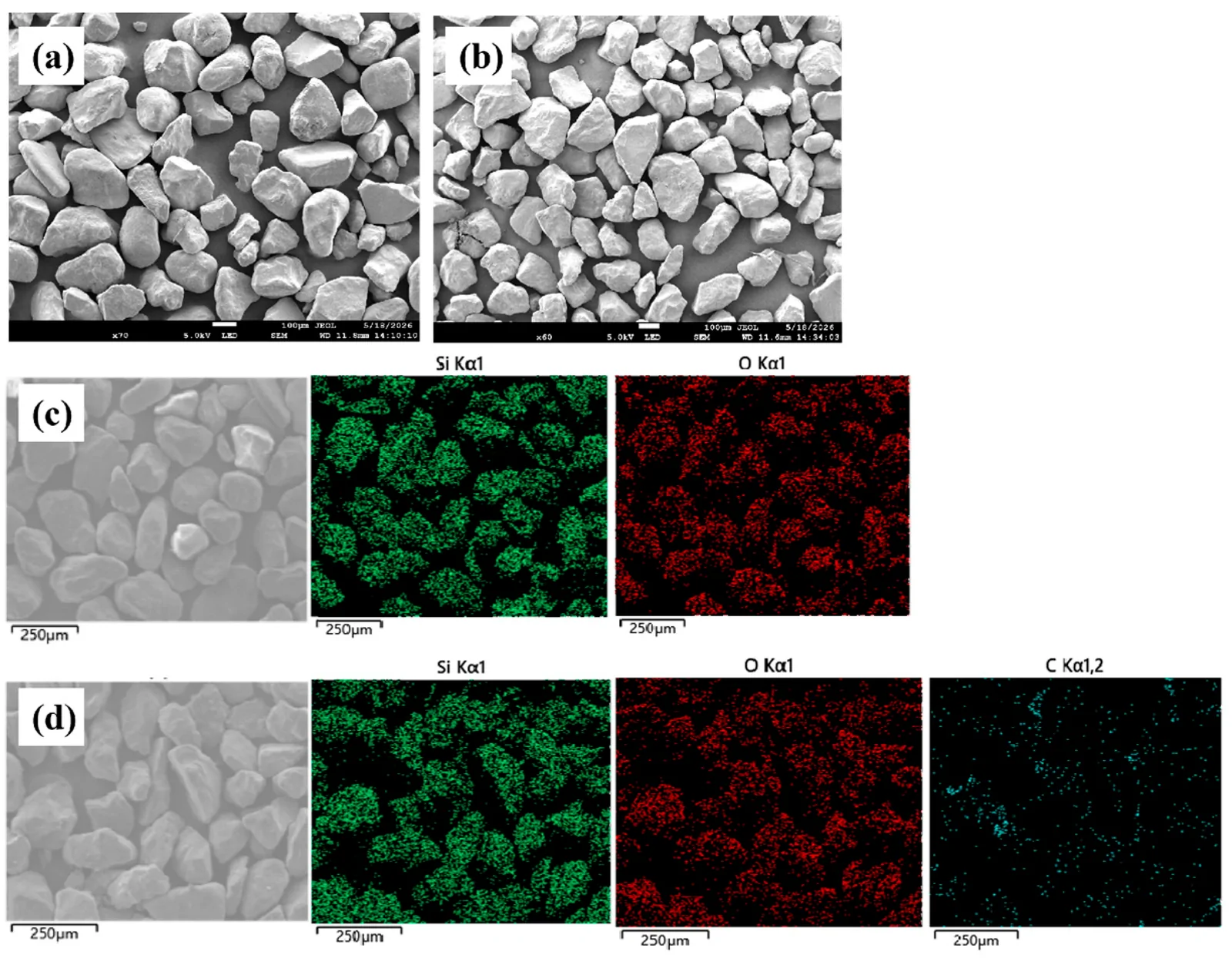
SEM micrographs of RQS (**a**) and MRQS (**b**); elemental mapping images of RQS (**c**) and MRQS (**d**).

Figure 3 shows the scanning electron microscope (SEM) microscopy and element distribution map of RQS and MRQS. SEM observation shows that the surface of RQS is bare and irregular, while the modified MRQS retains almost the same particle shape, confirming that the dry grafting process only changes the surface chemical composition and does not destroy the quartz skeleton. Elemental mapping showed that RQS displayed only Si and O signals, whereas continuous, homogeneous carbon signals entirely covered MRQS surfaces, evidencing uniform deposition of the organic functional layers. The even carbon distribution further confirms that high-speed shear mixing prevented local agglomeration of modifiers.

**Figure 4:**
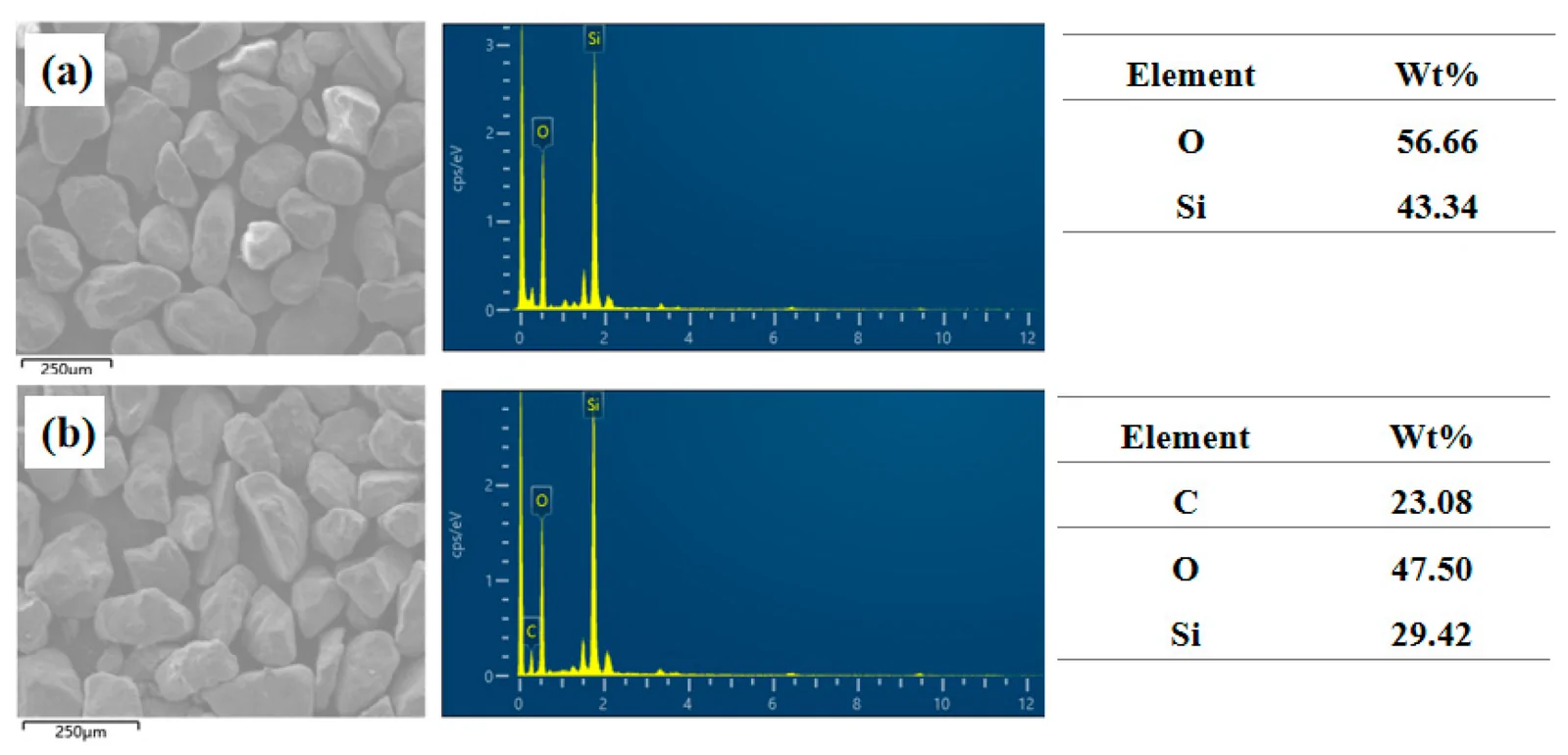
EDS spectra of RQS (**a**) and MRQS (**b**).

Figure 4 shows quantitative EDS spectra of RQS and MRQS. RQS contained only O (56.66 wt%) and Si (43.34 wt%) with negligible carbon. After modification, MRQS exhibited 23.08 wt% C, with decreased O (47.50 wt%) and Si (29.42 wt%). The attenuation of Si and O signals arises from shielding by the organic overlayer. Combined with elemental mapping, a compact, uniform organic film is confirmed to be covalently anchored on MRQS surfaces.

**Figure 5:**
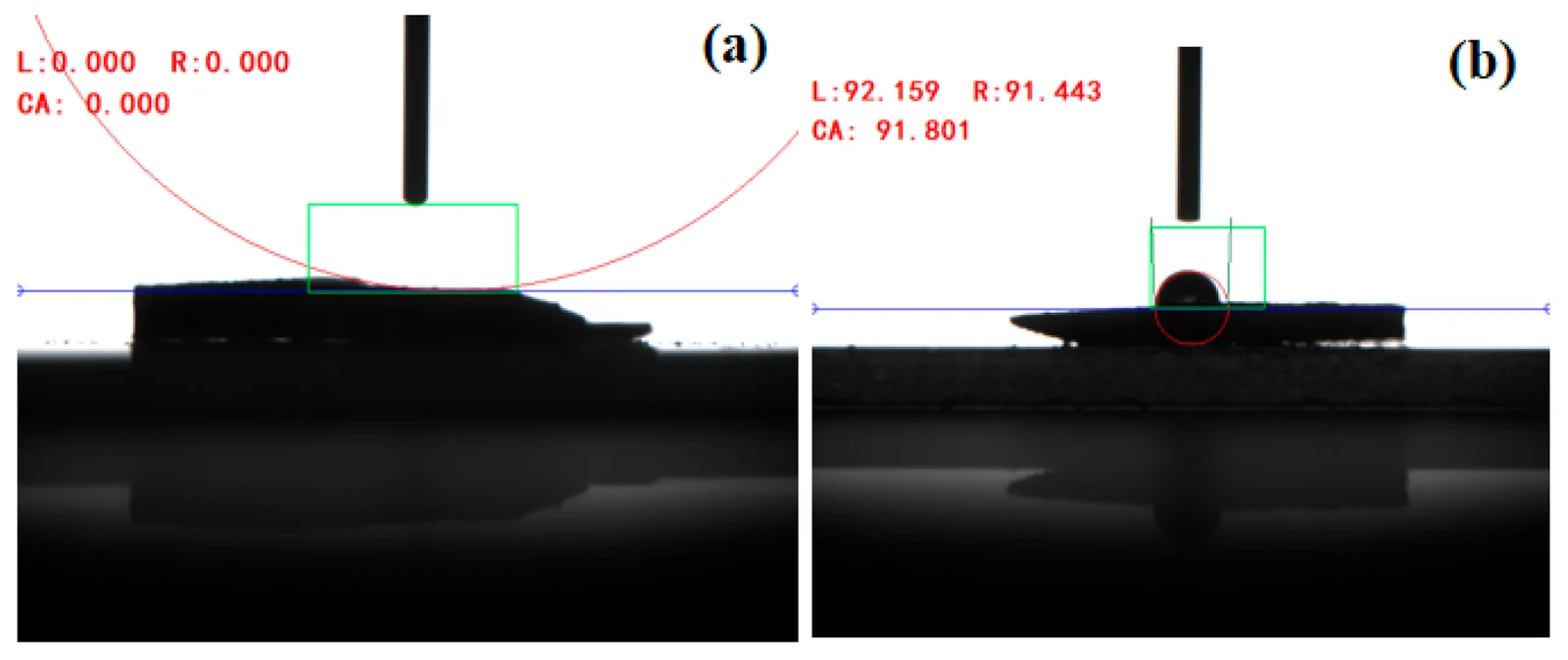
Contact angle (CA) images of RQS (**a**) and MRQS (**b**).

Figure 5 shows static water contact angle images. RQS exhibited a contact angle of approximately 0°, indicating strong intrinsic hydrophilicity. MRQS achieved a contact angle of 91.8°, demonstrating a dramatic wettability transformation induced by the hydrophobic alkyl and siloxane chains. This hydrophobic property is the core driving force for MRQS to spontaneously aggregate in water-based fracturing fluid.

**Figure 6:**
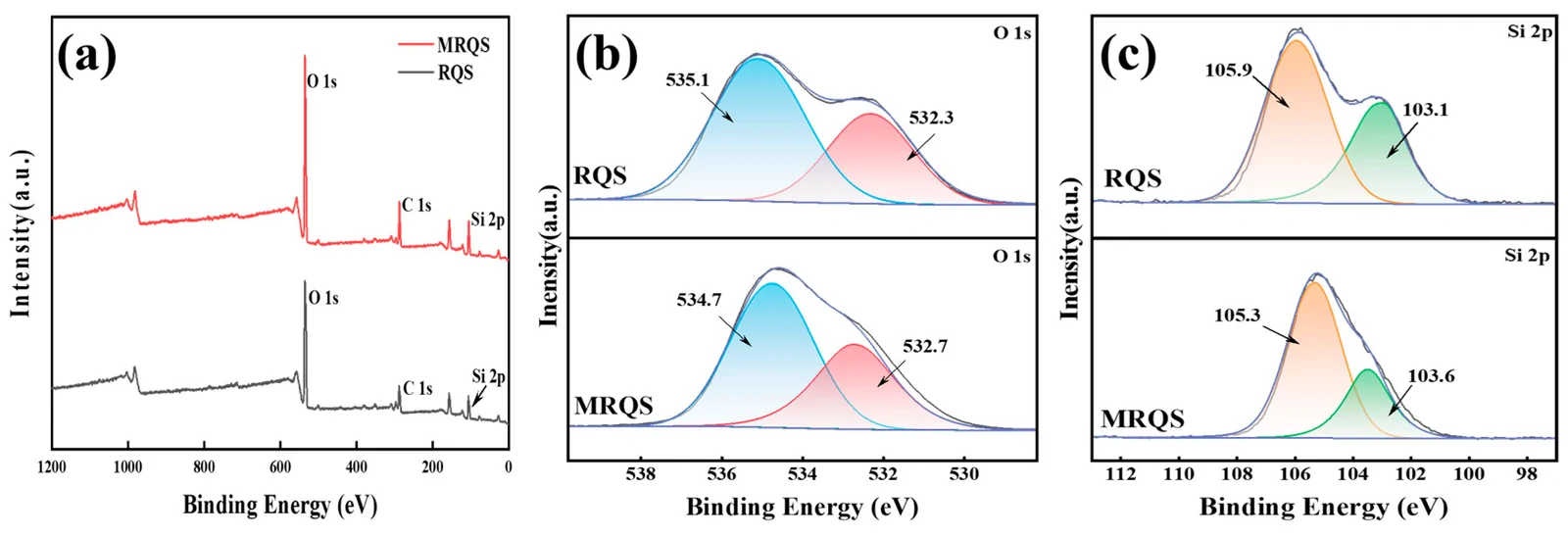
XPS spectra of RQS and MRQS. (**a**) Full-scan spectra, (**b**) O 1s spectra, and (**c**) Si 2p spectra.

Figure 6 shows the XPS spectrum of RQS and MRQS. For RQS, after deconvolutioning the Si 2p spectrum, two subpeaks are obtained: 103.1 eV (lattice Si) and 105.9 eV (defective Si site), while the O 1s spectrum is divided into 532.3 eV lattice oxygen and 535.1 eV hydroxyl oxygen. After organic coating, the energy levels of lattice Si and lattice oxygen move in the positive direction to 103.6 eV and 532.7 eV, while the defective Si and hydroxyl oxygen move to 105.3 eV and 534.7 eV in a negative direction, significantly reducing the energy gap between each pair. This convergence indicates that the organic film modulates surface potential and homogenizes the heterogeneous electronic structure of raw quartz surfaces. The enhanced C 1s signal in MRQS survey spectra further validates successful immobilization of organic modifiers.

**Figure 7:**
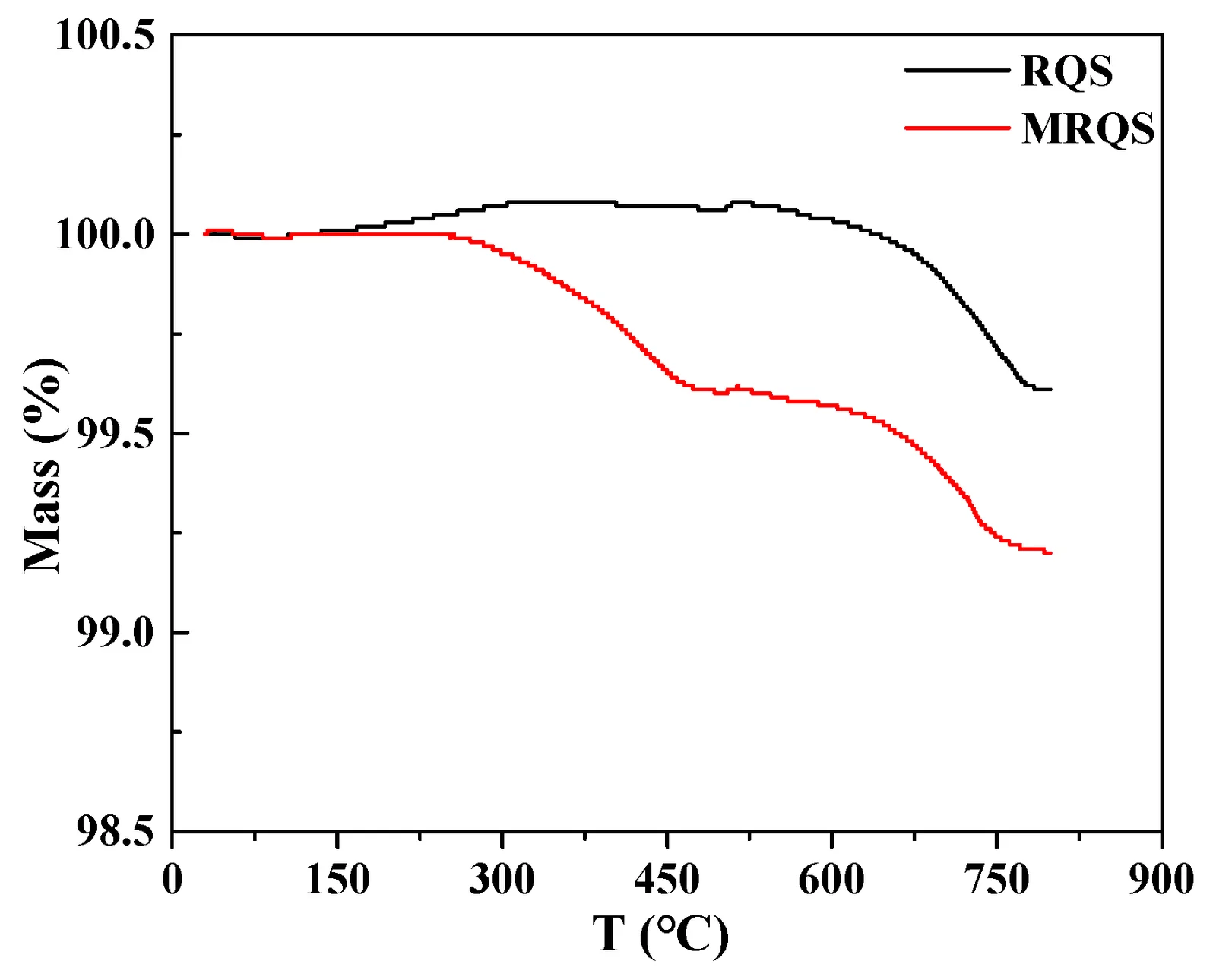
TGA curves of RQS and MRQS under N_2_ atmosphere.

Figure 7 shows the TGA curves of RQS and MRQS under air atmosphere. Raw quartz sand RQS maintains nearly constant mass from room temperature to 568 °C, demonstrating the excellent thermal stability of quartz substrate. Slight mass loss occurs above 568 °C, and the mass levels off at 784 °C with a residual mass of 99.60% of the initial value. This minor weight reduction is attributed to dehydroxylation on the quartz surface and thermal decomposition of trace inorganic impurities.

For modified quartz sand MRQS, obvious mass loss starts at 257 °C, corresponding to the thermal-oxidative decomposition of organic species on the sample surface. The major weight-loss interval of organic fractions ranges from 257 °C to 474 °C. A stable plateau is reached at 474 °C with a residual mass of 99.60%. This weight loss originates from oxidative volatilization of microcrystalline wax as well as oxidative degradation of organic chains of OTMS and H-PDMS. It should be noted that this mass loss includes both chemically bonded organic coating and a small amount of physically adsorbed organic species. The TGA curve remains stable from 474 °C to 586 °C, indicating that thermally oxidizable organic components have been almost completely consumed. A second slight mass-loss stage appears above 586 °C, which is consistent with the intrinsic thermal behavior of quartz substrate. The mass becomes constant after 775 °C, with a final residual mass of 99.20%.

The onset decomposition temperature of surface organic species is 257 °C, which is much higher than the typical reservoir temperature of CBM wells (below 120 °C). It demonstrates that MRQS possesses favorable thermal-oxidative stability under practical CBM reservoir thermal conditions.

### 3.2. Performance Evaluation Results

#### 3.2.1. Sand-Aggregation Performance

**Figure 8:**
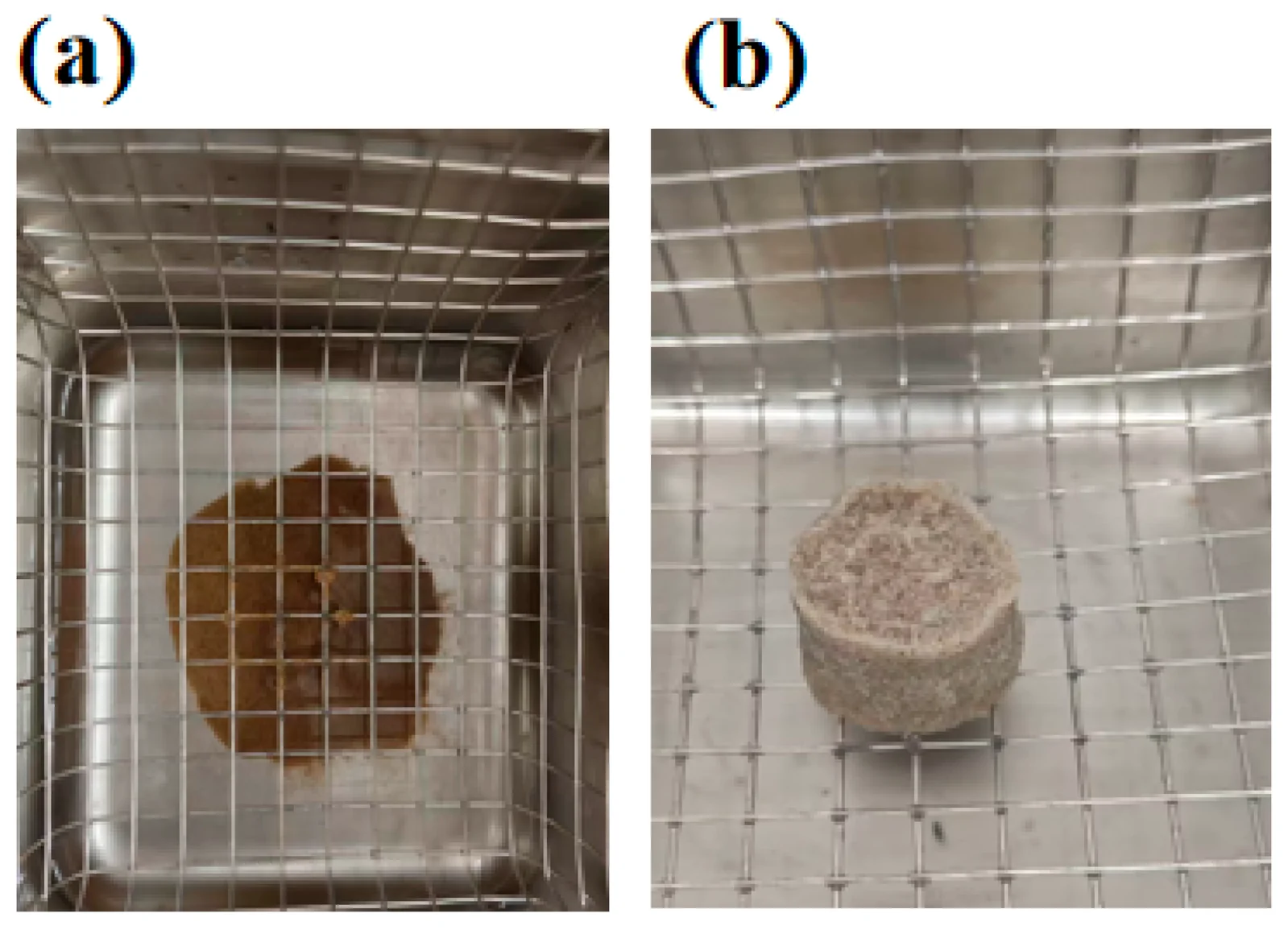
Morphological of RQS (**a**) and MRQS (**b**) aggregates after ultrasonic collapse test.

As summarized in Figure 8, obvious differences in the stability of sand columns constructed by RQS and MRQS were identified under ultrasonic oscillation. The RQS sand column fully collapsed within an average of 2.8 min, and fragmented particles penetrated through the metal mesh. By contrast, the MRQS sand column retained its complete morphology after 10 min of continuous ultrasonication and remained stably above the metal mesh. Raw quartz sand particles are merely stacked by weak physical interactions, whereas the functional coating on MRQS provides stable interfacial adhesion to form effective bridging between particles, greatly improving the structural strength of aggregates. Such excellent self-aggregation capacity can restrain proppant flowback and maintain stable proppant packing in fractures.

#### 3.2.2. Microscale Interfacial Adhesion and Morphology Analysis

**Figure 9:**
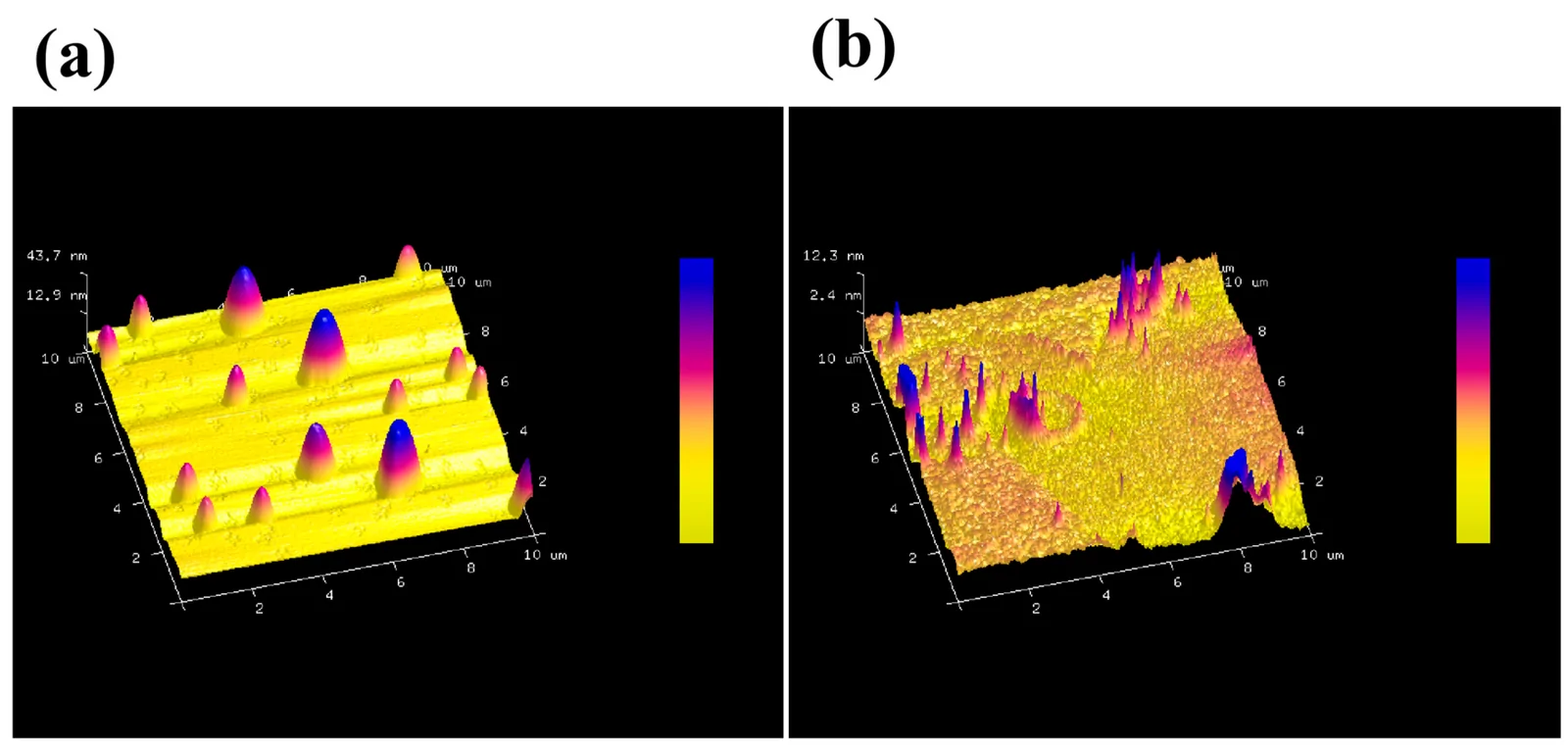
AFM topographic images of ball-milled RQS before (**a**) and after modification (**b**).

To interpret the distinct self-aggregation behavior observed in ultrasonic tests, AFM characterization (Figure 9) was conducted on ball-milled fine RQS and MRQS particles. Hence, the original 40–70 mesh quartz sand was ball-milled and sieved, and the obtained fine particles were modified following the identical coating protocol for bulk proppant samples. Despite the difference in particle size compared with the proppant adopted for macroscopic tests, the surface physicochemical characteristics induced by modification remain comparable. The average particle size increases from 0.76 μm (RQS) to 0.94 μm for MRQS, confirming that organic modifiers are successfully coated on quartz surfaces. Meanwhile, the average surface asperity height decreases from 50.5 nm (RQS) to 11.7 nm (MRQS). This phenomenon arises from microcrystalline wax within the composite modification system, which fills surface cavities and sharp protrusions of quartz particles and forms a smooth organic overlayer.

Interfacial adhesion forces under applied normal loads of 100 nN, 300 nN and 500 nN (Table 1) further reveal the particle interaction mechanism. For unmodified RQS, the adhesion force increased from 32.0 nN at 100 nN to 49.4 nN at 300 nN, which is in line with the trend predicted by JKR contact theory; that is, a larger load will expand the contact area and enhance the interface attractiveness. When the load further increases to 500 nN, interfacial slip and microdeformation cause the adhesion force to drop to 12.2 nN. Differently, MRQS achieves the highest adhesion force of 106.7 nN under 100 nN loading. The grafted hydrophobic organic coating provides strong intermolecular and hydrophobic interactions, supplying the essential driving force for spontaneous aggregation in aqueous fracturing fluid. As the normal load increases, the soft organic coating on the MRQS surface is gradually compressed and deformed, inducing molecular rearrangement of the surface functional layer. This structural change weakens the hydrophobic interfacial interaction, leading to a sequential decrease of adhesion force to 38.6 nN and 8.6 nN at 300 nN and 500 nN, respectively.

The low-load condition (100 nN) approximates the mild compressive state between adjacent proppant particles in underground fractures. The remarkably higher adhesion force of MRQS under low loading directly accounts for its superior aggregate stability and anti-flowback capacity, which is consistent with the sand-column ultrasonic collapse results described above.

#### 3.2.3. Temporary Plugging Performance

**Figure 10:**
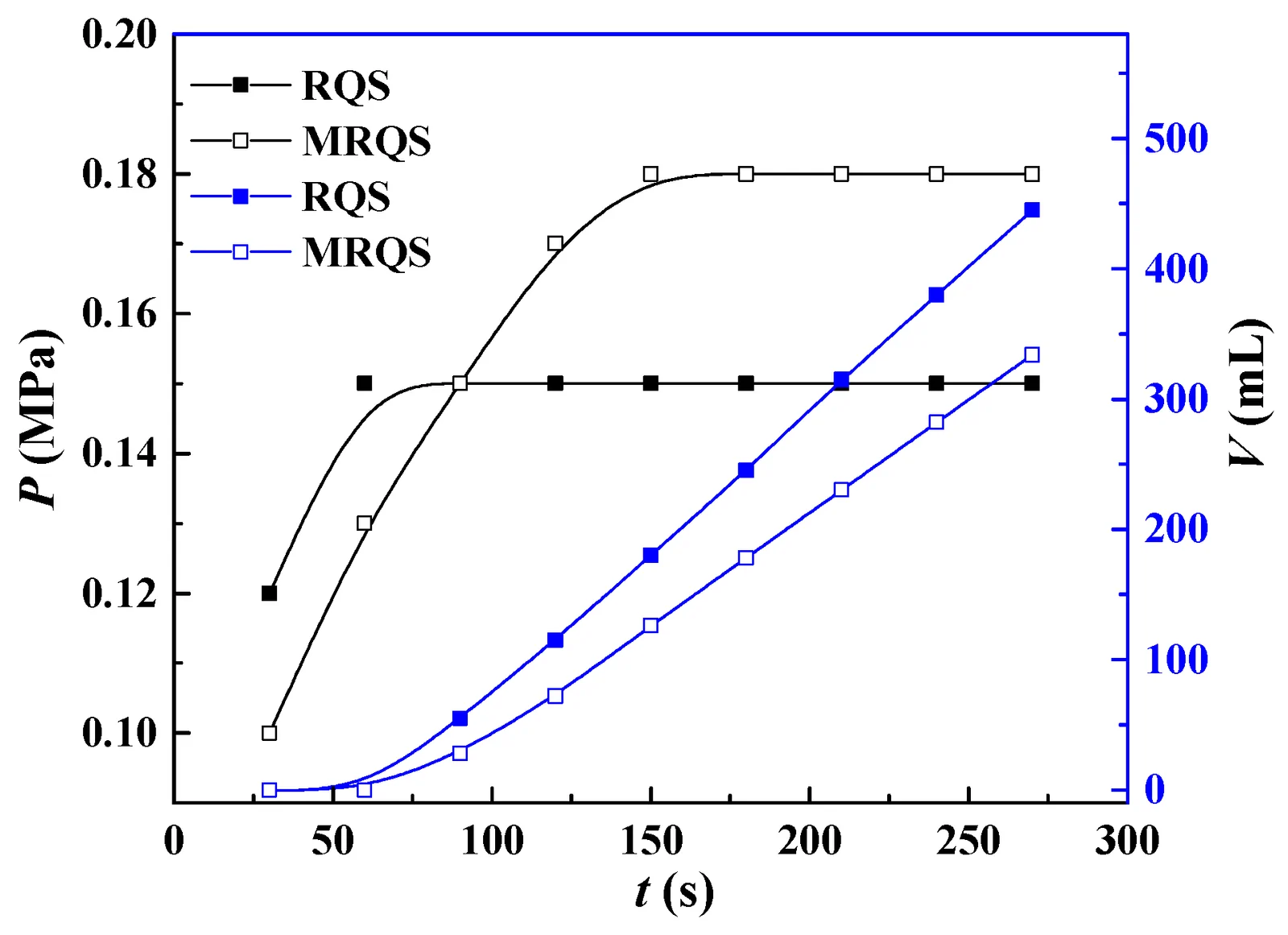
System pressure *P* and cumulative filtrate volume *V* of RQS and MRQS versus time *t*.

As shown in Figure 10, significant differences in pressure response and fluid loss were observed between RQS and MRQS. For RQS, the system pressure stabilized at only 0.15 MPa after 60 s with no rising tendency, while cumulative filtrate volume reached 445 mL at 270 s, indicating that RQS failed to form an effective plugging bridge at the fracture constriction. In contrast, MRQS exhibited steady pressure buildup from 0.11 MPa to a stable value of 0.23 MPa after 180 s, with drastically suppressed fluid penetration throughout. The 0.23 MPa temporary plugging pressure is suitable for temporary plugging and reconstruction of micro-fractures in shallow, low-pressure and low-permeability CBM reservoirs, while it is not applicable for high-pressure deep reservoir conditions, which defines the applicable boundary of MRQS plugging performance.

The hydrophobic organic layer endows MRQS with moderate interfacial adhesion, enabling spontaneous aggregation and compaction at the tapered fracture section (2 mm inlet, 1 mm outlet) to form a low-permeability plugging layer. Therefore, MRQS shows excellent compressive resistance and crack conduction performance.

#### 3.2.4. Fracture Conductivity Performance

**Figure 11:**
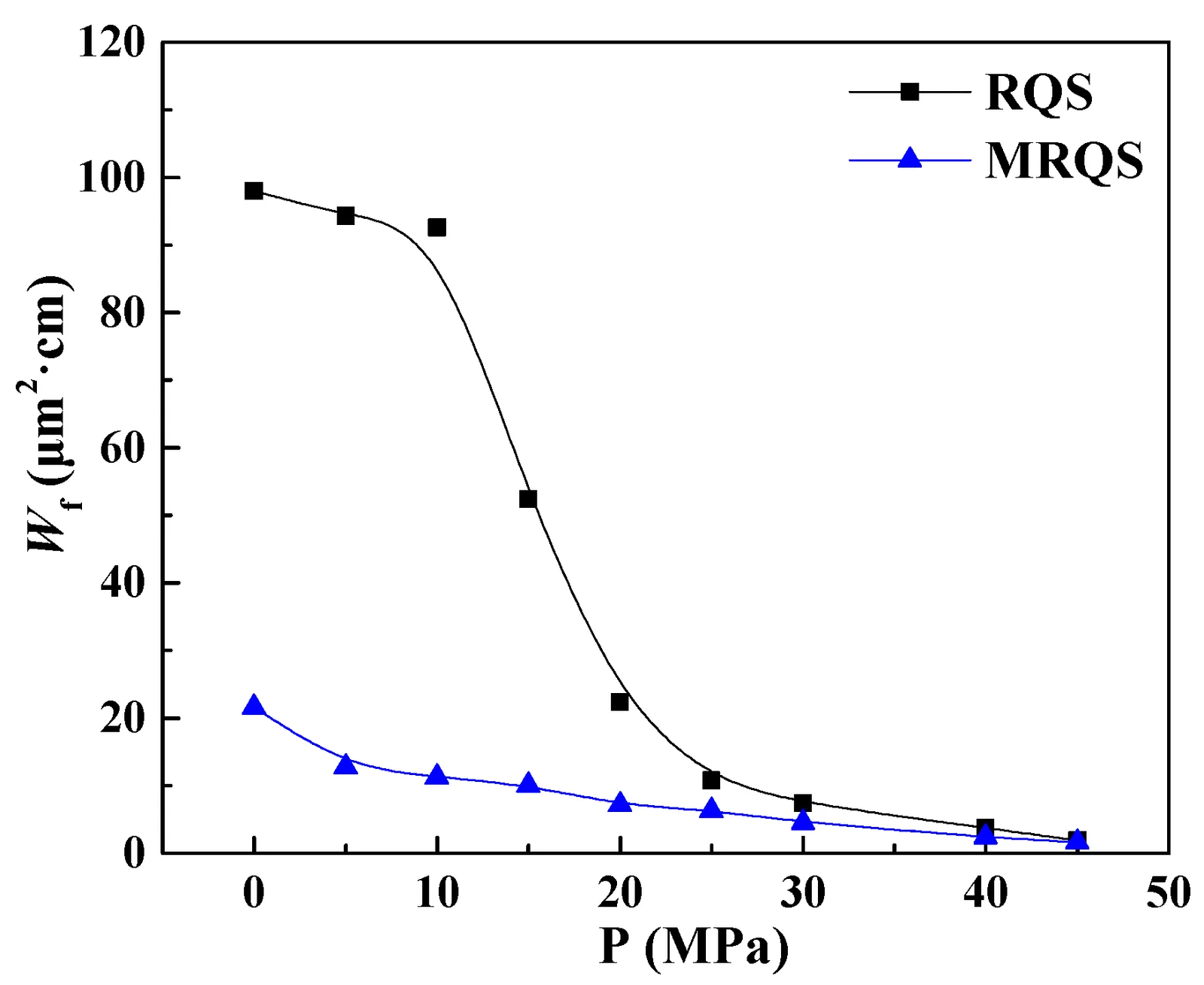
Fracture conductivity *W*_f_ of RQS and MRQS versus closure stress P.

As shown in Figure 11, the fracture conductivity W_f_ of both proppant packs declined monotonically with rising closure stress, with distinct differences in decay rate and absolute values. At 0–10 MPa, RQS exhibited much higher W_f_ (97.99–92.55 μm^2^·cm) than MRQS (21.62–11.30 μm^2^·cm), because the organic coating on MRQS triggered low-stress aggregation that filled primary pores. This reduction in low-stress conductivity is not only a side effect of modification but represents a targeted performance trade-off: high-conductivity RQS lacks inter-particle cohesion and delivers limited fracture-diversion capability, whereas moderately decreased conductivity of MRQS helps achieve local temporary plugging and stimulate new fracture networks for improved CBM recovery. Notably, MRQS still maintains effective fracture channels instead of complete flow blockage, balancing temporary-plugging function and fracture conductivity.

However, above 15 MPa, RQS suffered a steep decline to 1.88 μm^2^·cm at 45 MPa due to severe grain slip and pore collapse. In contrast, MRQS showed a smooth decay without sharp inflection, retaining 1.62 μm^2^·cm at 45 MPa. The moderate cohesion from self-aggregation formed mutually supported agglomerate skeletons that restrained pore compaction. MRQS thus exhibits superior anti-compaction performance under high in situ stress.

From the field perspective of coal-bed methane development in China, the closure stress of most coal reservoirs generally falls within the range of 0–15 MPa. Although RQS delivers higher fracture conductivity under low closure stress within this interval, it degrades drastically once stress exceeds 15 MPa. A considerable number of target CBM reservoirs in China are characterized by high in situ stress. In such high-stress coal-bed reservoirs, MRQS can maintain relatively stable fracture conductivity by forming self-supported agglomerate skeletons. Therefore, despite its relatively lower conductivity under low-stress conditions, MRQS exhibits more reliable long-term conductivity stability and is more suitable for high-stress CBM fracturing scenarios.

#### 3.2.5. Fluid-Loss Control Performance

**Figure 12:**
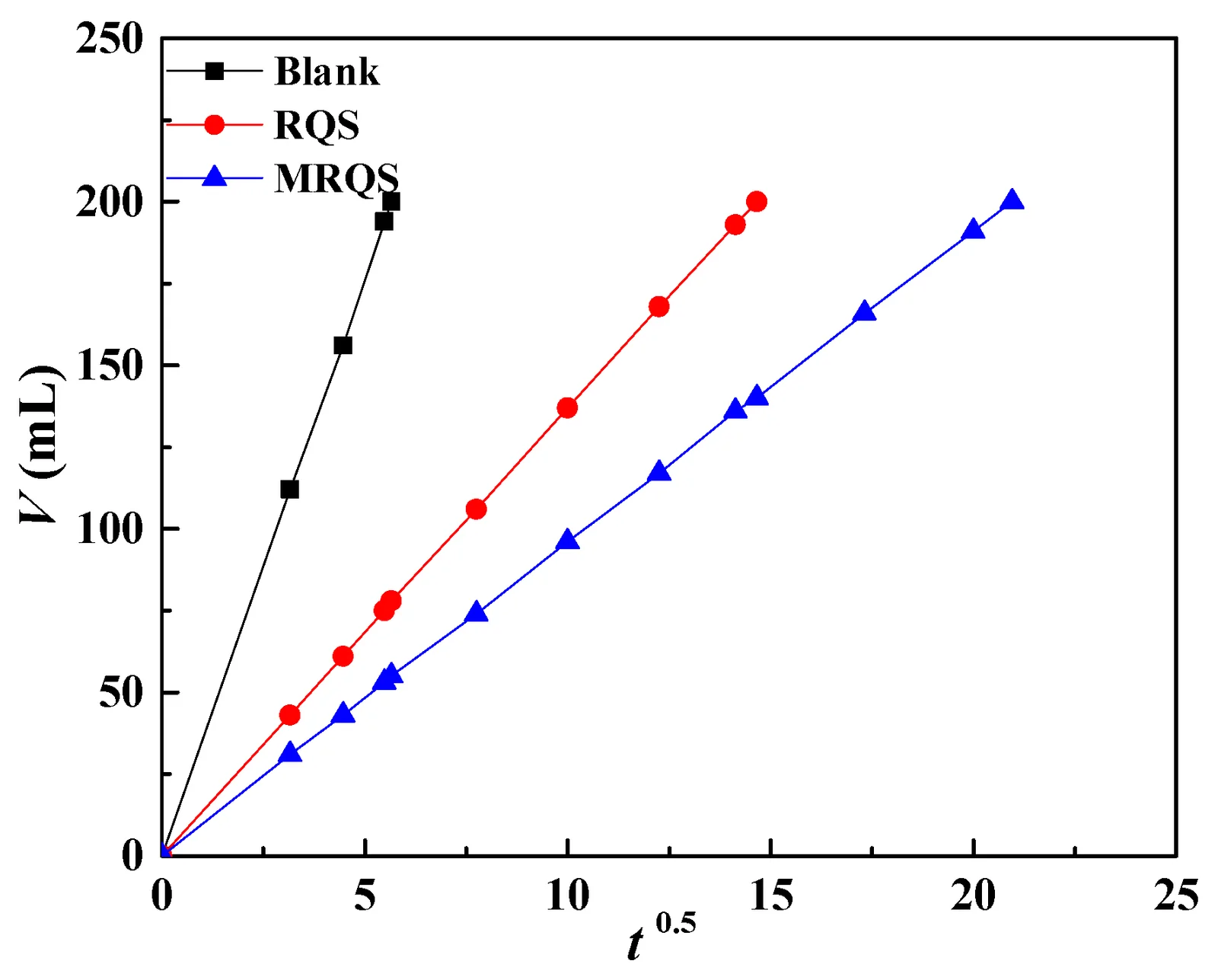
*V* − *t* ^0.5^ curves of three fracturing fluid systems.

Static fluid-loss performance of three fracturing fluid systems was evaluated following SY/T 6376. As summarized in Table 2 and Figure 12, the blank group (pure guar base fluid) exhibited the fastest fluid loss, reaching 200 mL at 32 s. Adding 30 vol.% RQS significantly suppressed fluid loss to 215 s for 200 mL, as inert quartz particles filled filter cake pores and increased seepage resistance. The MRQS group achieved optimal control, extending the filtration time of 200 mL to 439 s, which is about 2.04 times longer than that of the RQS group.

According to the constant pressure filtration theory (*V* = *k* · *t* ^0.5^), the order of fluid loss coefficient k is blank group > RQS group > MRQS group. The surface of the modified filler adsorbs the guar gum polymer chain, connects adjacent particles and fills the pores, thus forming a denser and low-permeability filter cake. This enhanced interface interaction fundamentally reduces the permeability of the filter cake and the invasion of the filtrate. By optimizing the filter cake structure and suppressing fluid loss, MRQS can also reduce water lock damage and the migration of coal powder to the matrix, achieving the dual benefits of fluid control and stratum protection, while maintaining good compatibility with the guar gum fracturing fluid system.

## 4. Conclusions

In this study, a multi-functional self-aggregation modified quartz sand support agent (MRQS) was successfully prepared by a simple dry coating technology for coal seam gas well channel fracturing. The organic coating transforms the surface of the raw sand from superhydrophilic to hydrophobic (contact angle 91.8°) without destroying the quartz skeleton. MRQS showed excellent self-aggregation stability (in the ultrasonic test, RQS was only 3 min, while MRQS was more than 10 min), which was attributed to the significant enhancement of its interface adhesion (106.7 nN at a load of 100 nN, while RQS was only 32.0 nN). The modified support agent can also form an effective temporary blockage layer (sealing pressure 0.23 MPa) at the crack contraction, and extend the static fluid loss time to 439 s, which is 2.04 times that of RQS.

The measurement results of fracturing conductivity show that MRQS can still maintain stable conductivity (1.62 μm^2^·cm) under 45 MPa closed stress, and the attenuation curve is smooth. Although the initial value is slightly lower than that of raw sand under low stress conditions, the performance is similar. It is worth noting that all tests in this work were carried out under simplified laboratory conditions with deionized water as the medium. The long-term coating durability, fracturing fluid compatibility, multiphase flow adaptability and coal fines interference resistance of MRQS under complex formation water and actual reservoir environments have not been systematically investigated. In addition, this study only focuses on the basic performance and modification mechanism of laboratory-prepared MRQS, and large-scale manufacturing consistency and economic evaluation need further exploration in future work.

In general, MRQS integrates spontaneous self-agglomeration, temporary blockage, leak-proof control and conductive stability under high stress into a single material, comprehensively solving the coupling problems faced by traditional support agents in the process of coal seam air fracturing. The simple dry coating process provides a facile and promising laboratory-scale preparation strategy for high-performance multifunctional proppants for CBM fracturing.

## Figures and Tables

**Table 1:** Interfacial adhesion forces of RQS and MRQS under different applied normal loads.

Applied Normal Load (nN)	Adhesion Force of RQS (nN)	Adhesion Force of MRQS (nN)
100	32.0	106.7
300	49.4	38.6
500	12.2	8.6

**Table 2:** Cumulative filtrate volume at different filtration times.

Time (s)	Time ^0.5^ (s ^0.5^)	Blank (mL)	RQS (mL)	MRQS (mL)
10	3.16	112	43	31
30	5.48	195	78	58
32	5.66	200	/	/
60	7.75	-	116	82
120	10.95	-	162	114
180	13.42	-	190	142
215	14.66	-	200	/
240	15.49	-	-	165
360	18.97	-	-	192
439	20.95	-	-	200

Note: “-” indicates test terminated upon reaching 200 mL.

## Data Availability

The original contributions presented in this study are included in the article. Further inquiries can be directed to the corresponding author.
